# *Brucella* Endocarditis as a Late Onset Complication of Brucellosis

**DOI:** 10.1155/2015/836826

**Published:** 2015-02-02

**Authors:** Panagiotis Andriopoulos, Christos Antoniou, Panagiota Manolakou, Athanassios Vasilopoulos, George Assimakopoulos, Maria Tsironi

**Affiliations:** ^1^Faculty of Nursing, University of Peloponnese, Orthias Artemidos & Plataion, 23100 Sparta, Greece; ^2^Department of Cardiology, Sparta General Hospital, 23100 Sparta, Greece; ^3^Department of Internal Medicine, Sparta General Hospital, 23100 Sparta, Greece

## Abstract

*Brucella* endocarditis (BE) is a rare but life threatening complication of *brucellosis*. We present a case report of a patient with relapsing brucellosis complicated with aortic valve endocarditis. The patient underwent valve replacement and required prolonged antibiotic treatment because of rupture of the noncoronary leaflet and development of congestive heart failure. Since the onset of endocarditis in patients with brucellosis is not known, proper follow-up is required in order to identify any late onset complications, especially in endemic areas.

## 1. Introduction

*Brucella* infection can affect different organs and systems and the disease course may be complicated by severe and/or rare life threatening clinical entities such as neurobrucellosis and endocarditis [1–3].* Brucella* endocarditis (BE) is one of the most challenging localizations of the disease, requiring prompt diagnosis and constant evaluation of the treatment plan in order to assess whether the patient will require surgical repair of the infected valve or continue with medical treatment alone [4–6]. However, little is known about the onset of endocarditis and possible measures to avoid this potentially fatal complication. Herein, we present a patient with relapsing* Brucella* infection complicated by* Brucella* endocarditis and we review the literature in order to clarify the specific characteristics of BE compared to other infectious causes of endocarditis.

## 2. Case Presentation

**Figure 1:**
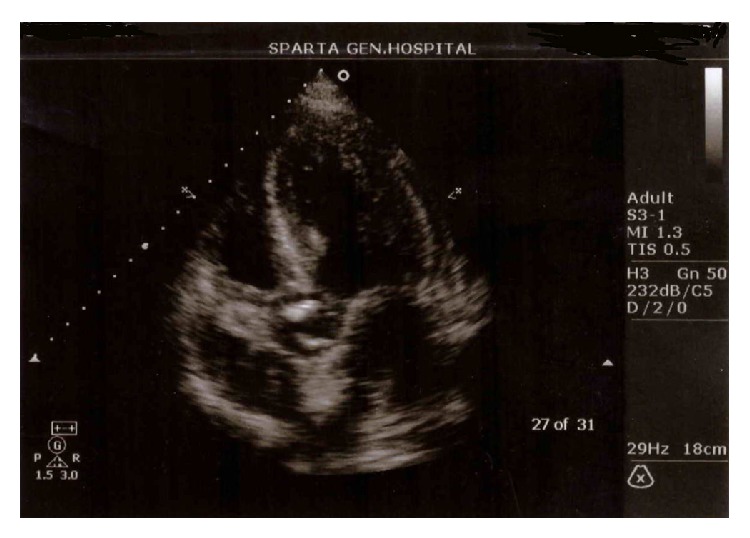
Transthoracic echocardiogram of the patient showing bicuspid aortic valve with vegetations.

A 50-year-old male livestock farmer was admitted to Sparta General Hospital with a 4-day history of fever, malaise, night sweats, and shortness of breath. He had been diagnosed with blood culture proven* Brucella* infection 6 months ago and was treated with a double regimen of 21 days of streptomycin and two months of doxycycline. He had recovered completely and* Brucella* antibodies titers were negative after two months of treatment. On admission, he was febrile (38.7°C) and physical examination revealed cervical lymphadenopathy, hepatosplenomegaly, and a 2/6 diastolic aortic murmur not reported in his previous medical history. According to CBC he was anemic (Ht 24%) and leukopenic (3.200/*μ*L), ESR was 102 mm/h, and biochemistry revealed a serum creatinine value of 2.1 mg/dL while BUN levels were 92 mg/dL. Wright standard tube agglutination test was positive in a titer of 1 : 1600. Blood cultures were positive for* Brucella* spp. that was identified later as* melitensis.* In transthoracic echocardiogram, vegetations were present on a bicuspid aortic valve (Figure 1). A triple antibiotic regimen with 900 mg of rifampicin, 200 mg of doxycycline daily, and streptomycin adjusted for renal failure was initiated. Transesophageal echocardiogram was performed the next day and confirmed the presence of active vegetations but also revealed severe aortic regurgitation and rupture of the noncoronary leaflet. He was referred for cardiothoracic consultation and a valve replacement surgery was decided. During hospitalization, fever, malaise, and renal failure had gradually resolved, but he developed decompensated heart failure requiring loop diuretics and oxygen supplementation. On the 21st day of treatment, streptomycin has been replaced with cotrimoxazole and three days later valve replacement was performed. The patient recovered without any surgical complications, continued antibiotic treatment for 6 months, and is currently well.

## 3. Discussion

Endocarditis is a well-documented complication of* Brucella* as far as clinical presentation and treatment options are concerned, accounting for the majority of fatal cases. A combined search of PubMed and Google Scholar for the terms “*Brucella*” and “endocarditis” in the title of the article returns 166 results, from as early as 1936. In two of the earliest reports Quintin and Stalker [7] and Hart et al. [8] describe the fatal outcome of* Brucella abortus* endocarditis. Aortic valve is the valve predominantly affected in* Brucella* endocarditis and mitral involvement is usually seen in preexisting rheumatic disease; preexisting valvular disease usually accounts for less than half of the reported cases in total [4, 9]. However healthy valves, as in this case report, also are often affected. Vegetations of the leaflets are the most common finding; however ulcerations, abscesses, and rupture of leaflets have been described [10, 11]. Congestive heart failure is the complication that may require emergency cardiac surgery. Keshtkar-Jahromi et al. [12] in a retrospective study of 308 cases assessed the role of surgery to the treatment and concluded that surgery reduced mortality of* Brucella* endocarditis from 32.7% in medical treatment alone to 6.7% in combined medical and surgical strategies (*P* < 0.001); similar results were reported in a study of 53 patients in Turkey [13].

*Brucella* is considered a rare endocarditis pathogen and is usually classified in culture negative endocarditis in review articles because* Brucella* spp. do not grow in usually used culture systems [14, 15]. However, the use of automated culture systems has reduced dramatically the time needed for identification of* Brucella* [16] and the term “culture negative” is not appropriate, since most reports have identified the pathogen in blood cultures [12, 13].

Whether BE is an acute infection or a result of chronic or relapsing* Brucella* infection is not clear. Recent reports on acute infectious endocarditis, using modern microbiology techniques, do not mention* Brucella* as a common pathogen [17, 18] but they come from nonendemic areas. In case series from endemic areas, patient history is often too vague to clarify whether endocarditis is an acute or late onset complication of the systemic infection [10, 12, 13, 19, 20].

In conclusion, we present a patient with late onset* Brucella* endocarditis that destroyed the noncoronary leaflet of his bicuspid aortic valve, requiring the combination of valve replacement and prolonged antibiotic treatment. The patient had a relapse of a previously successfully treated uncomplicated* Brucella* infection; this case report prompts the need of a proper follow-up of every* Brucella* infected patient after the initial diagnosis, since relapses may involve organs not affected in the first infection, especially in endemic areas and in individuals with professional exposure to risk factors for brucellosis.

## References

[1] Colmenero J. D., Reguera J. M., Martos F. (1996). Complications associated with Brucella melitensis infection: a study of 530 cases. *Medicine*.

[2] Bayer A. S., Bolger A. F., Taubert K. A. (1998). Diagnosis and management of infective endocarditis and its complications. *Circulation*.

[3] Franco M. P., Mulder M., Gilman R. H., Smits H. L. (2007). Human brucellosis. *The Lancet Infectious Diseases*.

[4] Reguera J. M., Alarcón A., Miralles F., Pachón J., Juárez C., Colmenero J. D. (2003). Brucella endocarditis: clinical, diagnostic, and therapeutic approach. *European Journal of Clinical Microbiology and Infectious Diseases*.

[5] Özsöyler I., Yilik L., Bozok Ş. (2005). *Brucella* endocarditis: the importance of surgical timing after medical treatment (five cases). *Progress in Cardiovascular Diseases*.

[6] Mert A., Kocak F., Ozaras R. (2002). The role of antibiotic treatment alone for the management of Brucella endocarditis in adults: a case report and literature review. *Annals of Thoracic and Cardiovascular Surgery*.

[7] Quintin T. J., Stalker M. R. (1946). Endocarditis due to *Brucella abortus*. *Canadian Medical Association Journal*.

[8] Hart F. D., Morgan A., Lacey B. (1951). Brucella abortus endocarditis. *British Medical Journal*.

[9] Cay S., Cagirci G., Maden O., Balbay Y., Aydogdu S. (2009). Brucella endocarditis—a registry study. *Kardiologia Polska*.

[10] Inan M. B., Eyileten Z. B., Ozcinar E. (2010). Native valve brucella endocarditis. *Clinical Cardiology*.

[11] Sasmazel A., Baysal A., Fedakar A. (2010). Treatment of Brucella endocarditis: 15 years of clinical and surgical experience. *Annals of Thoracic Surgery*.

[12] Keshtkar-Jahromi M., Razavi S.-M., Gholamin S., Keshtkar-Jahromi M., Hossain M., Sajadi M. M. (2012). Medical versus medical and surgical treatment for brucella endocarditis. *Annals of Thoracic Surgery*.

[13] Koruk S. T., Erdem H., Koruk I. (2012). Management of Brucella endocarditis: results of the Gulhane study. *International Journal of Antimicrobial Agents*.

[14] Baddour L. M., Wilson W. R., Bayer A. S. (2005). Infective endocarditis: diagnosis, antimicrobial therapy, and management of complications: a statement for healthcare professionals from the Committee on Rheumatic Fever, Endocarditis, and Kawasaki Disease, Council on Cardiovascular Disease in the Young, and the Councils on Clinical Cardiology, Stroke, and Cardiovascular Surgery and Anesthesia, American Heart Association: endorsed by the Infectious Diseases Society of America. *Circulation*.

[15] Habib G., Hoen B., Tornos P. (2000). Guidelines on the prevention, diagnosis, and treatment of infective endocarditis (new version 2009): the Task Force on the Prevention, Diagnosis, and Treatment of Infective Endocarditis of the European Society of Cardiology (ESC). Endorsed by the European Society of Clinical Microbiology and Infectious Diseases (ESCMID) and the International Society of Chemotherapy (ISC) for Infection and Cancer. *European Heart Journal*.

[16] Baysallar M., Aydogan H., Kilic A., Kucukkaraaslan A., Senses Z., Doganci L. (2006). Evaluation of the BacT/ALERT and BACTEC 9240 automated blood culture systems for growth time of Brucella species in a Turkish tertiary hospital. *Medical Science Monitor*.

[17] McDonald J. R. (2009). Acute infective endocarditis. *Infectious Disease Clinics of North America*.

[18] Murdoch D. R., Corey R. G., Hoen B. (2009). Clinical presentation, etiology, and outcome of infective endocarditis in the 21st century: the International Collaboration on Endocarditis-Prospective Cohort Study. *Archives of Internal Medicine*.

[19] Gunes Y., Tuncer M., Guntekin U. (2009). Clinical characteristics and outcome of *Brucella endocarditis*. *Tropical Doctor*.

[20] Cohen N., Golik A., Alon I. (1997). Conservative treatment for Brucella endocarditis. *Clinical Cardiology*.

